# Mitochondrial Toxicity of Azithromycin Results in Aerobic Glycolysis and DNA Damage of Human Mammary Epithelia and Fibroblasts

**DOI:** 10.3390/antibiotics8030110

**Published:** 2019-08-03

**Authors:** Xianpeng Jiang, Catherine Baucom, Robert L. Elliott

**Affiliations:** 1 Breast Cancer Research, Sallie A. Burdine Breast Foundation, Baton Rouge, LA 70806, USA; 2 Elliott Mastology Center, Baton Rouge, LA 70806, USA

**Keywords:** azithromycin, mitochondrial toxicity, aerobic glycolysis, DNA damage

## Abstract

Mitochondria evolved from free-living bacteria via endocytosis within eukaryotic host cells millions of year ago. We hypothesized that antibiotics cause mammalian mitochondrial damage while causing bacterial lethality. Mitochondrial toxicity of azithromycin in human mammary epithelia MCF-12A and fibroblasts were tested by fluorescent and transmission electron microscopy. Gene expression and DNA damage were tested by real-time polymerase chain reaction (qPCR) and ELISA. We found azithromycin suppressed the mitochondrial membrane potential gradient of MCF-12A cells and fibroblasts. Ultrastructure exams showed that the antibiotic caused vacuolated and swollen mitochondria with disrupted cristae in MCF-12A cells and fibroblasts compared to the morphology of mitochondria in the cells without antibiotic treatment. Fluorescent microscopy also showed azithromycin-induced mitochondrial reactive oxygen species (ROS), superoxide, after 3 h of culture. The DNA oxidative damage product, 8-hydroxy-2’-deoxyguanosine (8-OHdG, significantly increased in the media after MCF-12A cells and fibroblasts were cultured in the media containing azithromycin for 24 h. Azithromycin upregulated gene expression of hypoxia inducible factor 1 alpha (*HIF1a*), glycolytic enzymes including hexokinase 2 (*HK2*), phosphofructokinase 1 (*PFKM*), pyruvate kinase muscle isozyme M2 (*PKM2*), and glucose transporters in MCF-12A cells and fibroblasts. Lactate production also increased in the culture media. After treatment with azithromycin, healthy MCF-12A and fibroblast cells increased aerobic glycolysis—the “Warburg Effect”—to generate energy. In summary, azithromycin caused mitochondrial toxicity, ROS overproduction, DNA oxidative damage, upregulation of the *HIF1a* gene, and aerobic glycolysis in healthy mammalian cells. Over-usage of antibiotics could contribute to tumorigenesis and neurodegeneration and aggravate existing mitochondria-associated diseases.

## 1. Introduction

Azithromycin binds to the 23S rRNA of the bacterial 50S ribosomal subunit. It blocks protein synthesis by inhibiting the transpeptidation/translocation step of protein synthesis and by inhibiting the assembly of the 50S ribosomal subunit [1]. It has bacteriostatic properties against a wide spectrum of both Gram-positive and Gram-negative bacteria, atypical bacteria, and some protozoa. Special pharmacokinetic properties of azithromycin include a wide antimicrobial spectrum, long half-life, excellent tissue penetration and extensive tissue distribution, and high drug concentrations within cells. The most prominent feature of azithromycin is its ability to concentrate in intracellular compartments, mainly in fibroblasts, hepatocytes, phagocytic cells, and white blood cells. Patients who take 500 mg of azithromycin orally have peak plasma concentrations (C_max_) of 0.21–0.54 µg/mL on Day 1, and tissue concentrations may reach 1000 times higher than plasma concentrations [2,3,4,5].

Mitochondria evolved from free-living bacteria via endocytosis within eukaryotic host cells millions of year ago. Mitochondria share similar ribosomes and protein synthesis machinery as do bacteria [6,7]. Thus, it is inevitable that antibiotics cause mammalian mitochondrial damage while causing bacterial lethality. 

Azithromycin is well tolerated and common side effects of azithromycin include upset stomach, diarrhea/loose stools, nausea, vomiting or abdominal pain [8]. Serious incidents include hearing loss and QT prolongation, the latter which may result in death in specific circumstances. Hearing loss has been observed in a diminutive percentage of individuals, but there are case reports to confirm this severe side effect after short-term administration of oral azithromycin [9]. The cardiac side effects may be associated with mitochondrial toxicity of azithromycin. Salimi et al. [10] reported azithromycin-induced reactive oxygen species (ROS) formation, mitochondrial membrane permeabilization, and mitochondrial swelling and cytochrome c release in cardiomyocyte mitochondria of rats. The cardiomyocyte mitochondrial toxicity of azithromycin might be a starting point for cardiac side effects of azithromycin, such as QT prolongation, torsades de pointes, and arrhythmia in patients [10]. In rats, azithromycin also induced swelling of the mitochondria of hepatocytes and Browicz–Kupffer cells [11]. 

In the present study, we used clinically similar tissue concentrations of azithromycin to treat a human mammary epithelial cell line and primary fibroblasts. The mitochondrial ultrastructure and membrane potentials, gene expression of mitochondrial oxidative phosphorylation (OXPHOS) and glycolytic enzymes, mitochondrial ROS, and DNA damage were examined. The main objective of this report was to emphasize that azithromycin can cause mitochondrial dysfunction and DNA damage in human cells.

## 2. Results

### 2.1. Azithromycin Inhibited the Proliferation of MCF-12A and Fibroblast Cells

**Figure 1:**
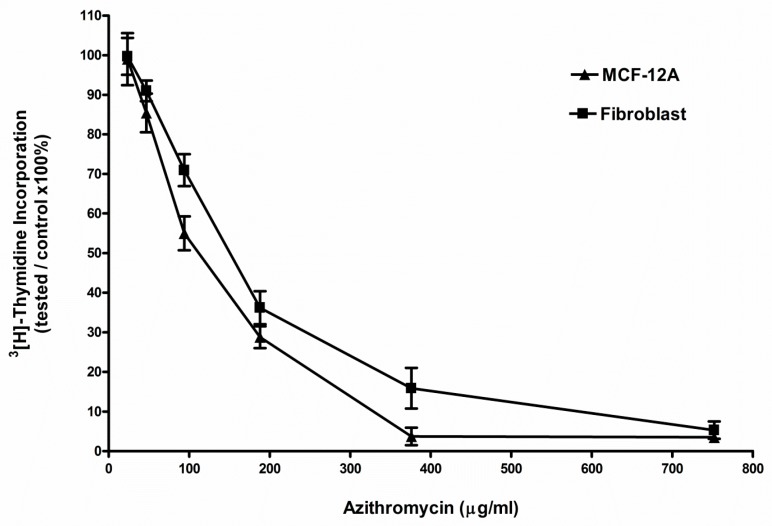
Azithromycin inhibited the proliferation of MCF-12A cells and fibroblasts in a dose-dependent pattern. Cells were incubated in the media containing azithromycin for 7 days.

The ^3^(H)-thymidine incorporation assay showed that azithromycin inhibited the proliferation of MCF-12A and fibroblast cells in a dose-dependent manner (Figure 1). The 50% inhibitory concentrations (IC_50_) of azithromycin were 94 ± 33 µg/mL and 115 ± 49 µg/mL in MCF-12A and fibroblast cells, respectively, after 7 days of azithromycin treatment. 

### 2.2. Azithromycin Suppressed Mitochondrial Membrane Potential Gradient

**Figure 2:**
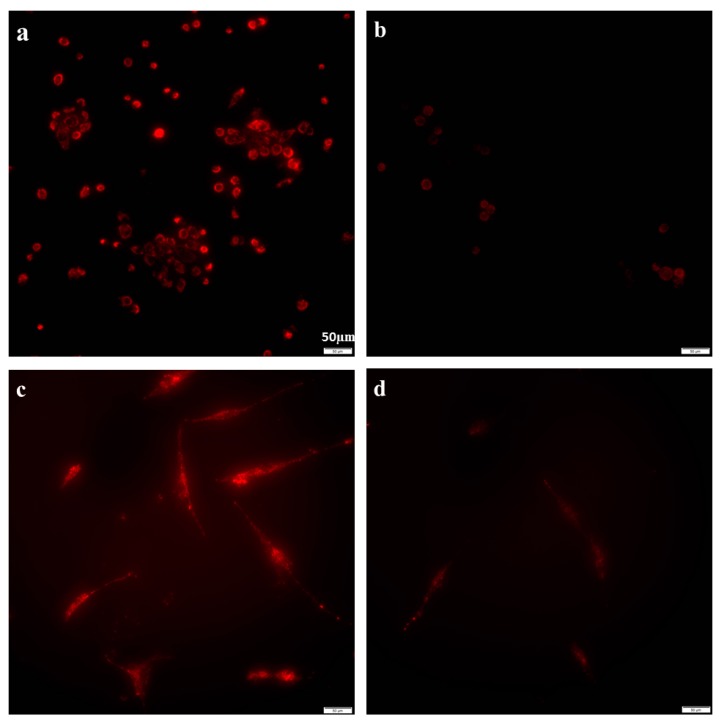
Azithromycin suppressed the mitochondrial membrane potential of MCF-12A cells and fibroblasts (20×). Mitochondria were stained with JC-1. (**a**) MCF-12A control; (**b**) MCF-12A treated with 188 µg/mL azithromycin for 24 h; (**c**) fibroblast control; (**d**) fibroblasts treated with 188 µg/mL azithromycin for 24 h.

The MCF-12A and fibroblast cells were treated with 188 µg/mL of azithromycin for 24 h. Mitochondrial potential was detected by 5,5′,6,6′-tetrachloro-1,1′,3,3′-tetraethylbenzimidazolocarbocyanine iodide (JC-1) staining. The mitochondrial membrane potential of the MCF-12A cells was significantly inhibited by treatment with 188 µg/mL of azithromycin [fluorescent intensity (FI): 20.2 ± 8.9] compared to the control cells (56.5 ± 12.5) (*p* < 0.05) (Figure 2a,b), and 188 µg/mL of azithromycin also significantly suppressed the mitochondrial membrane potential of the fibroblasts (FI: 35.5 ± 7.9) compared to the control cells (55.3 ± 5.2) (*p* < 0.05) (Figure 2c,d). 

### 2.3. Mitochondrial Ultrastructure Damage by Azithromycin

**Figure 3:**
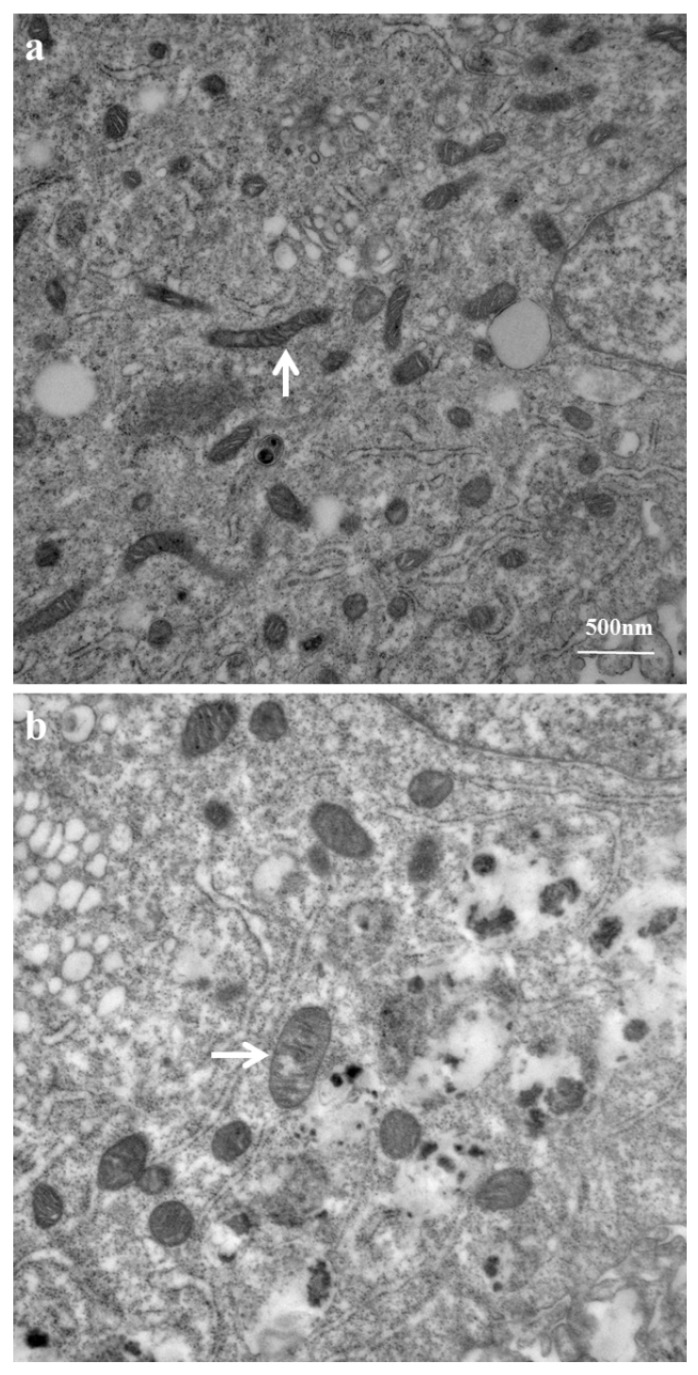
Azithromycin caused swollen and vacuolated mitochondria in MCF-12A cells. (**a**) control; (**b**) azithromycin 94 µg/mL for 7 days; arrow: mitochondria.

The MCF-12A cells were treated with 94 µg/mL of azithromycin for 7 days. The ultrastructure of the cells was examined by transmission electron microscopy. We found that azithromycin caused vacuolated and swollen mitochondria with disrupted cristae in the MCF-12A cells compared to the normal structure of mitochondria in the cells without azithromycin treatment (Figure 3). 

### 2.4. Azithromycin Upregulated Expression of Mitochondrial OXPHOS, Mitochondrial Biogenesis, and Antioxidant Genes

**Figure 4:**
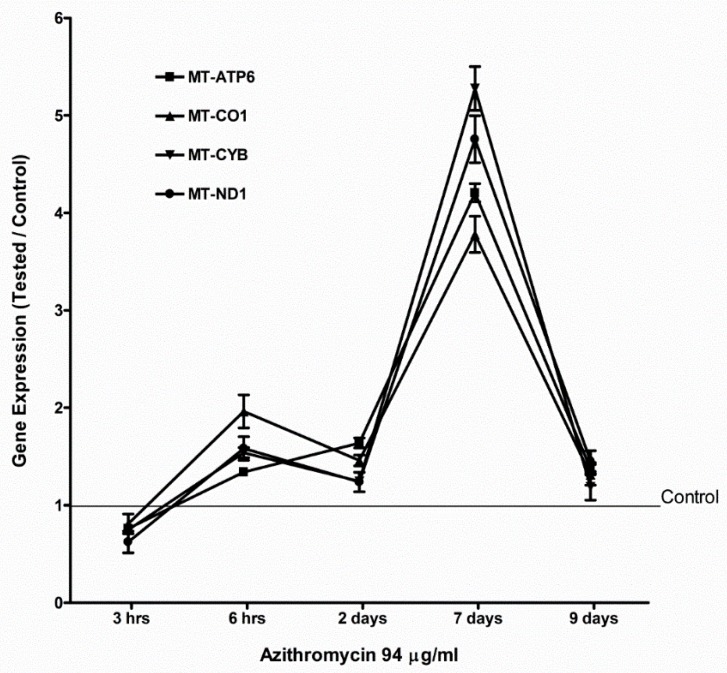
Azithromycin upregulated gene expression of mitochondrial OXPHOS enzymes in MCF-12A cells. *MT-ATP6*: mitochondrially encoded ATP synthase 6; *MT-CO1*: mitochondrially encoded cytochrome c oxidase 1; *MT-CYB*: mitochondrially encoded cytochrome b; *MT-ND1*: mitochondrially encoded NADH dehydrogenase 1.

**Figure 5:**
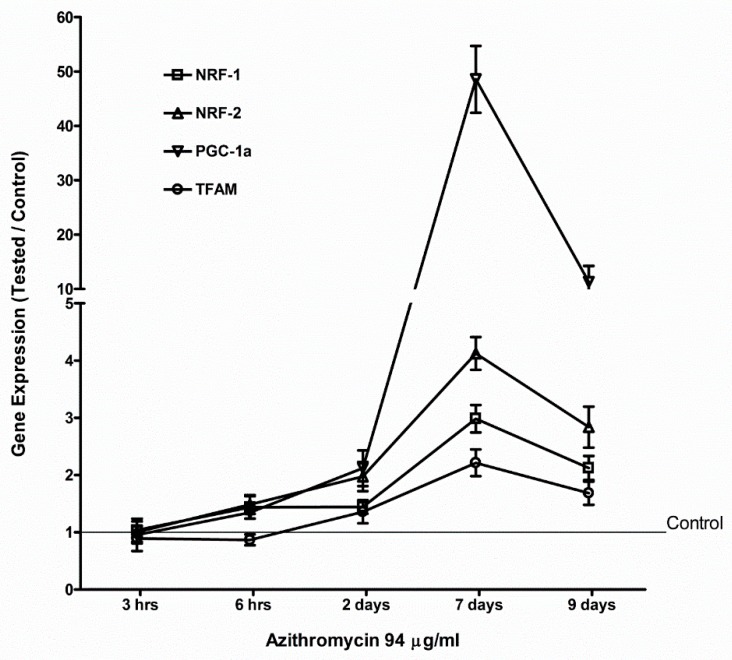
Azithromycin increased gene expression of mitochondrial biogenesis factors in MCF-12A cells. *NRF-1*: nuclear respiratory factor 1; *NRF-2*: nuclear respiratory factor; *PGC-1a*: peroxisome proliferator-activated receptor-gamma coactivator 1 alpha; *TFAM*: mitochondrial transcription factor A.

**Figure 6:**
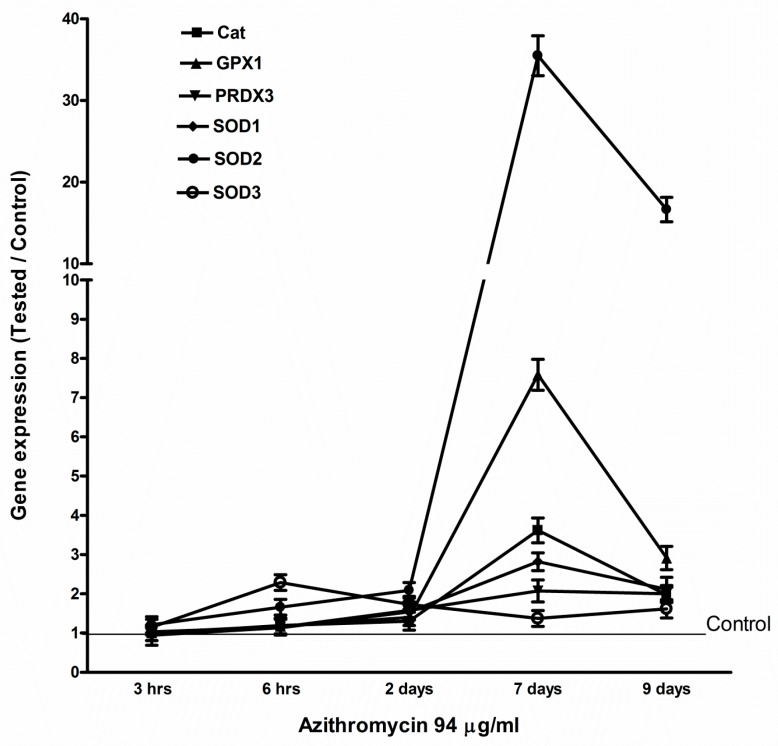
Azithromycin upregulated the expression of antioxidant genes in MCF-12A cells. *Cat*: catalase; *GPX1*: glutathione peroxidase 1; *PRDX3*: peroxiredoxin 3; *SOD1*, -*2*, -*3*: superoxide dismutase 1, 2, 3.

The MCF-12A cells were treated with 94 µg/mL of azithromycin. Total RNA was isolated at 3 h, 6 h, 2 d, 7 d, and 9 d. The levels of mRNA were measured by qPCR. Even though azithromycin primarily targets ribosomes to inhibit protein synthesis, azithromycin slightly suppressed the gene expression of mitochondrial OXPHOS enzymes, mitochondrially encoded ATP synthase 6 (*MT-ATP6*), mitochondrially encoded cytochrome c oxidase 1 (*MT-CO1*), mitochondrially encoded cytochrome b (*MY-CYB*), and mitochondrially encoded NADH dehydrogenase 1 (*MT-ND1*) in MCF-12A cells at the early stage (3 h). Then, the gene expression of mitochondrial OXPHS enzymes increased and peaked at day 7, returning to base level at day 9 (Figure 4). Azithromycin also upregulated the expression of mitochondrial biogenesis and antioxidant genes. The expression of mitochondrial biogenesis and antioxidant genes reached a peak at day 7 and returned to base levels at day 9 (Figure 5 and Figure 6). The peroxisome proliferator-activated receptor-gamma coactivator 1 alpha (*PGC1a*) gene was the most sensitive mitochondrial biogenesis gene to azithromycin and the *PGC1a* mRNA increased almost 50 times that of the control at day 7. Superoxide dismutase 2 (*SOD2*) and glutathione peroxidase 1 (*GPX1*) were the two major antioxidant genes responding to azithromycin. The effects of 115 µg/mL of azithromycin on the expression of mitochondrial OXPHOS and the mitochondrial biogenesis and antioxidant genes of fibroblasts were similar to that of MCF-12A cells (Figure S1). These data suggest MCF-12A cells and fibroblasts started cell repairing mechanisms in response to azithromycin.

### 2.5. Azithromycin-Induced Gene Expression of HIF1a, Glycolytic Enzymes, and Glucose Transporters

**Figure 7:**
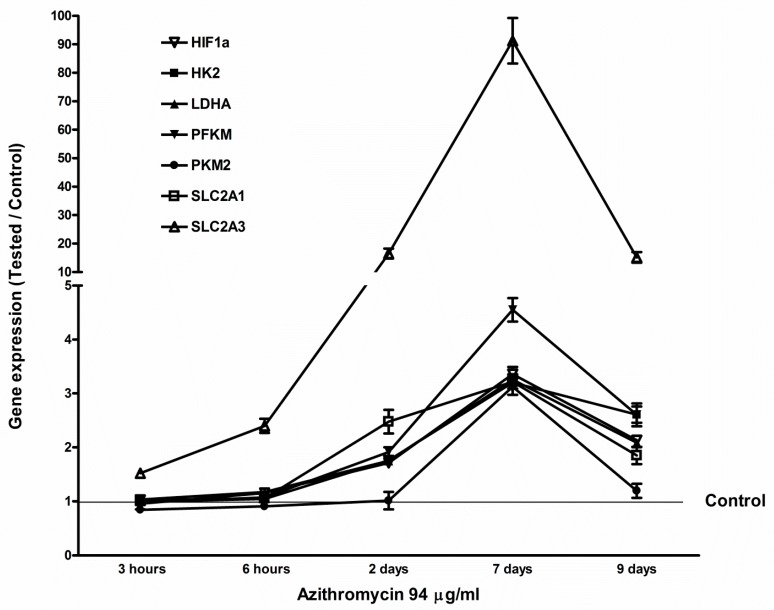
Azithromycin increased gene expression of *HIF1a* and glycolysis-associated enzymes in MCF-12A cells. *HIF1a*: hypoxia-inducible factor 1 alpha; *HK2*: hexokinase; *LDHA*: lactate dehydrogenase A; *PFKM*: phosphofructokinase-1; *PKM2*: pyruvate kinase; *SLC2A1*: glucose transporter 1; *SLC2A3*: glucose transporter 3.

The MCF-12A cells and fibroblasts were treated with 94 µg/mL and 115 µg/mL of azithromycin (concentration of IC_50_), respectively. Total RNA was isolated at 3 h, 6 h, 2 d, 7 d, and 9 d. The controls were cultured in the media without any antibiotic. Gene expression was measured by qPCR. Azithromycin upregulated the expression of hypoxia-inducible factor 1 alpha (*HIF1a*), hexokinase (*HK2*), phosphofructokinase-1 (*PFKM*), pyruvate kinase (*PKM*), lactate dehydrogenase A (*LDHA*), glucose transporter 1 (*SLC2A1*), and glucose transporter 3 (*SLC2A3*) in MCF-12A cells. The mRNA levels reached a peak at 7 days of treatment, then gradually decreased to base levels (Figure 7). The effects of azithromycin on gene expression of *HIF1a, HK2, PFKM, PKM, LDHA, SLC2A1*, and *SLC2A3* in fibroblasts were similar to that of MCF-12A cells (Figure S1). These results suggest that azithromycin induces aerobic glycolysis of mammary cells.

### 2.6. Azithromycin Increased Lactate Production

The MCF-12A cells and fibroblasts were cultured with azithromycin in media for 24 h. The concentrations of lactate in media were significantly increased in human mammary epithelium MCF-12A cells (*p* < 0.05) compared to MCF-12A cells cultured in medium without any antibiotic. Azithromycin also increased the lactate concentrations in the culture medium of fibroblasts, the difference was significant statistically (*p* < 0.05) (Table 1).

### 2.7. Azithromycin Induces Mitochondrial Superoxide

**Figure 8:**
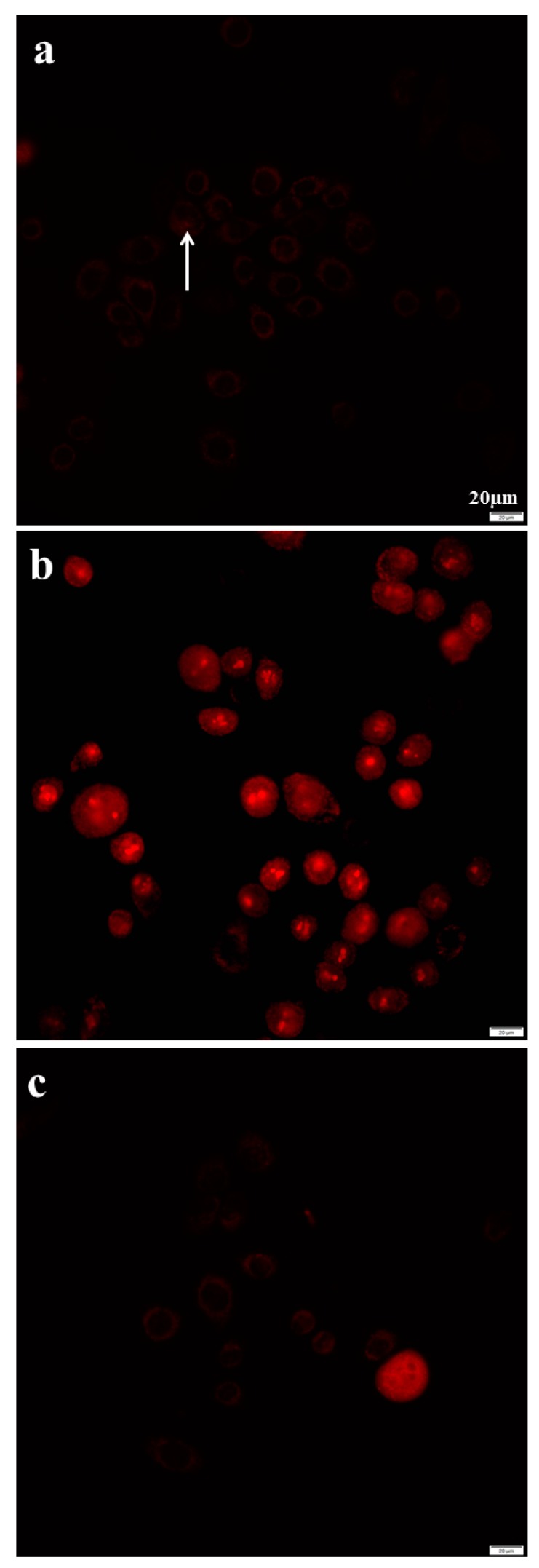
Azithromycin increased mitochondrial ROS of MCF-12A cells. Antioxidant SOD could prevent the production of ROS. (**a**) control; (**b**) 188 µg/mL of azithromycin for 3 h; (**c**) 188 µg/mL azithromycin + 100 u/mL SOD for 3 h; arrow: ROS.

The predominant reactive oxygen species (ROS) was superoxide. The mitochondrial superoxide was determined by the MitoSOX red mitochondrial superoxide indicator. The intensity of fluorescence was measured by the software Cellsens 1.16. The addition of 188 µg/mL of azithromycin in media induced mitochondrial superoxide production of MCF-12A cells after 3 h of culture (Figure 8). The fluorescent intensity significantly increased in MCF-12A cells and fibroblasts compared to the controls cultured in media without azithromycin (Table 2). We also found that superoxide dismutase (SOD) could prevent mitochondrial superoxide production induced by azithromycin when 100 u/mL of SOD was added to the media with 188 µg/mL azithromycin (Figure 8c and Table 2).

### 2.8. Azithromycin Causes Cell DNA Oxidative Damage

**Figure 9:**
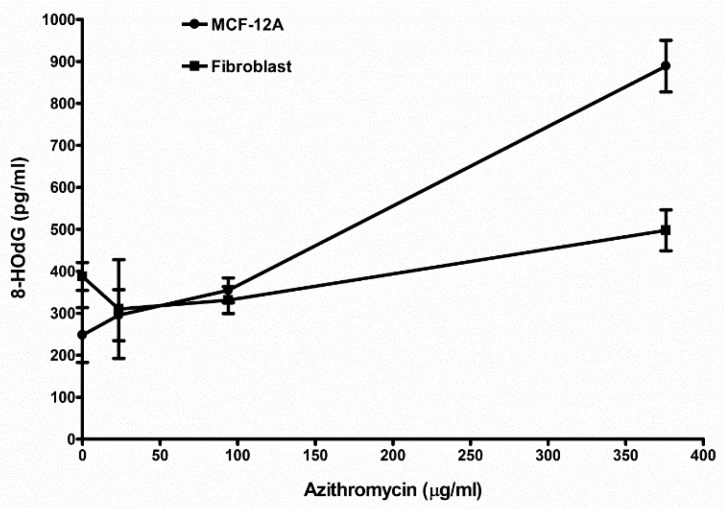
Azithromycin induced oxidative DNA damage in MCF-12A cells and fibroblasts. The details can be seen in the Materials and Methods section.

The MCF-12A cells and fibroblasts were cultured in media containing azithromycin for 24 h. The concentrations of 8-OHdG in culture media were measured. Azithromycin induced 8-OHdG product in the media in a dose-dependent pattern (Figure 9, Table 3). These results show azithromycin causes DNA oxidative damage of human cells MCF-12A and fibroblast.

## 3. Discussion

It is believed that antibiotics specifically target bacteria; however, the consequences of how they interact with mammalian cells have largely been overlooked. According to endosymbiotic theory, mitochondria originated from free-living aerobic bacteria [12]. It is possible that antibiotics target mitochondria and mitochondrial components similar to bacteria. Azithromycin inhibits the bacterial 50S ribosome [1] In the present study, ultrastructural changes confirmed that azithromycin caused vacuolated and swollen mitochondria with disrupted cristae in human mammary epithelial MCF-12A cells. Azithromycin also inhibited the proliferation of MCF-12A cells and fibroblasts in a dose-dependent pattern. These results confirmed azithromycin can target mammalian mitochondria and cause mitochondrial dysfunction. Antibiotic adverse effects include gastrointestinal stress, ototoxicity, retinopathy, chondrotoxicity, photosensitivity, anaphylactoid reaction, nephrotoxicity, etc. All of these side effects are likely to have unique etiologies. However, as new evidence is revealed, mitochondrial alterations form the basis for various adverse effects with chemically distinct drugs. Inhibitors of prokaryotic ribosomes, gyrases, and topoisomerases can induce unexpected effects on mitochondria that lead to side effects of these antibiotics. Inhibition of protein synthesis is associated with many of the mitochondrion-based toxicities [13,14]. Kalghagti et al. [15] reported that clinically relevant doses of bactericidal antibiotics, ciprofloxacin (a fluoroquinolone), ampicillin (a β-lactam) and kanamycin (an aminoglycoside), induced a dose- and time-dependent increase in intracellular ROS production and the DNA oxidative damage product 8-OHdG in the human immortalized, untransformed mammary epithelia cell line MCF-10A [15]. In mammalian cells, mitochondria are major sources of intracellular ROS. Superoxide (O_2_^•−^) is the predominant mitochondrial ROS. Murphy [16] stated there are three modes of mitochondria operation that produce superoxide in isolated mitochondria. In the first mode, the NADH pool, is reduced by damage to the respiration chain. This leads to a rate of O_2_^•−^ formation at the Flavin mononucleotide (FMN) of complex I. In the second mode, there is no ATP production and there is a high proton-motive force (ΔP) and reduced coenzyme Q (CoQ) pool which leads to reverse electron transport (RET) through complex I, producing large amounts of O_2_^•−^. In the third mode, mitochondria are actively making ATP. The rate of O_2_^•−^ production is far lower than in modes 1 and 2, and the sites of production are uncertain. Thus, mitochondria mainly produce ROS during damage to respiration and no ATP production. In bacteria, antibiotics induced toxic ROS via the disruption of the tricarboxylic acid (TCA) cycle and the electron transport chain (ETC) [17]. Kalghagti et al. [15] found that bactericidal antibiotics inhibited mitochondrial ETC complexes, in particular complexes I and III, which have been identified as major sources of ROS formation [15,18]. In the present study, azithromycin suppressed gene expression of mitochondrial OXPHOS enzymes at 3 h (Figure 4) and mitochondrial respiration with decreased mitochondrial membrane potential at 24 h (Figure 2). Azithromycin also induced mitochondrial ROS after 3 h of culture (Figure 8). These results suggested that azithromycin induced ROS by suppression of mitochondrial respiration. The elevated ROS led to DNA oxidative damage, with increased 8-OHdG production in the media in a dose-dependent pattern in MCF-12A cells and fibroblasts cultured with azithromycin for 24 h. The qPCR showed that gene expression of antioxidants started to increase at 2 days and reached peak levels. The upregulation of antioxidants appears to be too late to protect mammalian cells from ROS damage. Many previous studies showed ROS damage was associated with diseases such as tumorigenesis and neurodegeneration [19,20]. In Gulam Waris and Haseeb Ahsan’s [19] review, they state that ROS influences central cellular processes such as proliferation, apoptosis, and senescence which are implicated in the development of cancer. Azithromycin is able to concentrate in intracellular compartments, mainly in fibroblasts, hepatocytes, phagocytic cells, and white blood cells, where concentrations reach up to 321 µM (240 µg/mL), approximately 1000 times that of C_max_. Shimura et al. [21] reported that mitochondrial ROS of myofibroblasts induced by radiation activated TGF_β_ signaling and promoted tumor growth. Numerous studies provide evidence that chronic inflammation increases the risk of cancer [22,23]. Chronic inflammation is mostly caused by infectious agents. Thus, those patients often take long-term antibiotic treatment. Mitochondrial toxicity of antibiotics may contribute to their carcinogenesis. The risk factor of antibiotics in carcinogenesis is often neglected in the medical community. One statistical study showed that the use of antibiotics was associated with increased risk of incident and fatal breast cancer. Although further studies are needed, their findings suggest a cautious long-term evaluation of antibiotic use [24]. 

Mitochondria play an important role in ageing-related neurodegenerative disorders such as Parkinson’s disease, Alzheimer’s disease, Huntington’s disease, and amyotrophic lateral sclerosis. Mitochondrial respiratory suppression, mitochondrial DNA damage, and increased ROS induced apoptosis, protein misfolding, and proteasomal malfunction of neural cells [20]. We found that azithromycin inhibited mitochondrial membrane potential and induced mitochondrial superoxide in mouse motor neuronal NSC-34 cells after 2 h of culture (Figure S2). These preliminary results suggested long-term antibiotic usage might contribute to neurodegeneration. Patients with neurodegeneration often suffer from infections and are treated with antibiotics more than healthy people. The antibiotic usage will worsen the neurodegeneration. In our current study, superoxide dismutase (SOD) prevented mitochondrial superoxide production induced by azithromycin (Figure 8). The addition of antioxidant SOD might protect cells from oxidative damage by azithromycin. More studies remain to be done. This suggests neurodegenerative patients should take antioxidants when they have to be treated with antibiotics. 

Gene expression of *HIF1a* and *PGC1a* was upregulated after MCF-12A cells and fibroblasts were treated with azithromycin. This is healthy cells’ compensatory response to suppression of mitochondrial respiration and overproduction of mitochondrial ROS. HIF1a induces glycolytic metabolism in hypoxia and normoxia [25,26]. After azithromycin treatment, gene expression of glycolytic enzymes, including *HK2, PFKM, PKM,* and *LDHA*, and glucose transporters *SLC2A1* and *SLC2A3*, were upregulated. Cells make ATP from glycolysis and produce more lactate than cells without antibiotic treatment. *HIF1a* also promotes oncogenes and plays an important role in carcinogenesis [27,28,29]. *PGC1a* promotes mitochondrial biogenesis and respiration [30], and can also potentially reduce generation of mitochondrial driven ROS. *PGC1a* is required for the induction of many ROS-detoxified enzymes including GPx1 and SOD2 [31,32]. In the present study, gene expression of *PGC1a* increased approximately 50 times by day 7 after cells were incubated with 94 µg/mL of azithromycin. Thus, *PGC1a* is a very important factor for mitochondrial and cell health. Other mitochondrial biogenesis-associated genes *NRF1*, *NRF2,* and *TFAM* were also upregulated. Coincidentally with *PGC1a*, gene expression of antioxidants started to increase at day 2 and reached peak levels at day 7 after azithromycin treatment. These results showed cells started a self-defense mechanism from mitochondrial ROS induced by azithromycin; however, defense was much slower than ROS production. We found increased ROS and DNA damage at 3 h after azithromycin treatment. Gene defects and variation of antioxidant enzymes have been reported in diabetic patients with nephropathy, patients with red blood cell disorders, and infertile men [33,34,35]. Those individuals may be vulnerable to antibiotic toxicity. 

Many cell lines are incubated in media containing antibiotics, such as gentamicin, penicillin or streptomycin, to prevent bacterial contamination. We found that gentamicin addition in routine culture media caused mitochondrial toxicity, increased mitochondrial ROS and changed cell metabolism [36]. There are many papers in the literature on the effects of antibiotics on metabolism of cell lines. Unfortunately, many of the experiments were performed in cell culture media containing antibiotics, while the investigators were testing the effect of another antibiotic on cell metabolism. Those previous results may need to be repeated in antibiotic-free culture media. In the present study, all cell lines were maintained in antibiotic-free media. 

In summary, azithromycin with clinically comparable concentrations induced mitochondrial toxicity, ROS production, and DNA damage in the human mammary epithelial line MCF-12A and primary fibroblast. Azithromycin also upregulated *HIF1a* and glycolysis-associated genes and induced aerobic glycolysis of the cells. Healthy cells started their defense systems such as upregulation of genes of mitochondrial biogenesis and antioxidants. However, this defense was much slower than ROS damage. In clinical practice, we suggest that patients take antioxidant agents while they are being treated with antibiotics, especially in patient groups with chronic infection, neurodegeneration, and genetic mitochondrial diseases. 

## 4. Materials and Methods

### 4.1. Cell Culture

The human immortalized, untransformed mammary epithelia cell line MCF-12A was obtained from American Type Culture Collection (Rockville, MD, USA). The MCF-12A cells were cultured in a 1:1 mixture of Ham’s F12 medium and Dulbecco’s Modified Eagle’s Medium containing 0.1 µg/mL cholera enterotoxin, 10 µg/mL insulin, 0.5 µg/mL hydrocortisone, 20 µg/mL epidermal growth factor, and 5% horse serum (Sigma Chemical Co., St. Louis, MO, USA). Fibroblast was derived from female adult connective tissue; 250 mg of surgically removed connective tissue was minced with sterile scalpels and digested for 5 h at 37 °C in alpha-MEM containing 6.25% fetal calf serum (GIBCO Invitrogen, Carlsbad, CA, USA), 0.56% collagenase (Worthington Biomedical, Lakewood, NJ, USA), and 0.004% DNase (Sigma–Aldrich, St. Louis, MO, USA). Then, the cell mixture was centrifuged for 5 min at 400× *g* and the supernatant removed. The cell pellet was re-suspended in completed alpha-MEM supplemented with 10% fetal calf serum and 1 mM glutamine (GIBCO Invitrogen, Carlsbad, CA, USA). When large fibroblast colonies appeared, the fibroblast colonies were removed with a sterile cell scraper from the flask and transferred to a new flask to culture. When the fibroblasts grew to 80% full in the flask, they were digested with 0.05% trypsin and 0.53 mM EDTA (GIBCO Invitrogen, Carlsbad, CA, USA). All cell lines were maintained in media without supplement of antibiotics such as gentamicin, penicillin, streptomycin, etc.

### 4.2. ^3^(H)-Thymidine Incorporation Assay

Six thousand MCF-12A cells or fibroblasts per well in 100 µL media were plated in 96 well culture plates and incubated overnight at 37 °C. Then, the media were aspirated and replaced with fresh media containing azithromycin. The control was replaced with media without any antibiotic. The plate was continuously cultured for another 4 days. The ^3^(H)-thymidine (0.1 µCi/well, MP Biomedical, Santa Ana, CA, USA) was added to all wells for the last 16 h of incubation. The cells were removed from the plates by trypsin–EDTA digestion, and harvested onto a glass-fiber filter (Skatron Basic 96 Harvester, Skatron, Inc., Sterling, VA, USA). The filters were placed into scintillation fluid, and the radioactivity was counted by liquid scintillation (LS 1800, Beckman Co., Fullerton, CA, USA). Cell proliferation was quantitated by ^3^(H)-thymidine incorporation and expressed as a percentage of the control. All the ^3^(H)-thymidine incorporation experiments were done in triplicate and were repeated three times.

### 4.3. Mitochondria Staining for Mitochondrial Membrane Potential

Ten thousand MCF-12A cells or fibroblasts were cultured in 35 mm × 1.5 mm glass bottom dishes containing antibiotic-free media overnight. The media were aspirated on the second day. The dishes were filled with media containing 188 µg/mL azithromycin (99% purity, purchased from Sigma Chemical Co., St. Louis, MO, USA). The control was filled with media only. All cells were then cultured for 3 h at 5% CO_2_ and 37 °C. Mitochondria were stained with mitochondria staining kit (Sigma CS0390, St. Louis, MO, USA). The brief procedure was as follows: Mix 25 µL of 200× JC-1 Stock Solution in 4 mL of ultrapure water in a test tube. Close the test tube and mix the solution by inversion or vortex test tube briefly. Incubate the test tube for 2 min at room temperature to ensure that the JC-1 is completely dissolved. Open the test tube and add 1 mL of JC-1 Staining Buffer 5×. Mix by inversion. Then, mix the staining mixture with an equal volume of complete medium for cell growth. Aspirate growth medium from flask and overlay cells with the above mixture. Add 0.2–0.4 mL of the mixture per 1 cm^2^ of growth surface. Incubate cells for 20 min at 37 °C in humidified atmosphere containing 5% CO_2_. Finally, aspirate the mixture and wash the cells twice with cold growth medium. Fluorescence was observed by an Olympus IX83 fluorescent microscope (Tokyo, Japan). In cells which maintained an electrochemical potential gradient, the dye concentrated in the mitochondria where it formed bright red fluorescent aggregates (J-aggregates). Three pictures were taken in different areas in each dish. All pictures were taken with 400 ms exposure. Fluorescent intensity (FI) was measured with the software Cellsens 1.16 and the mean and standard deviation of the intensity was calculated.

### 4.4. Mitochondrial Ultrastructure Examination

The MCF-12A cells were treated with 94 µ/mL azithromycin for 7 days at 37 °C. The control MCF-12A cells were cultured in medium without any antibiotics. Cells were digested with 0.05% trypsin-EDTA and pelleted by centrifugation. Cell pellets were fixed with a solution containing 3% glutaraldehyde and 2% paraformaldehyde in 0.1 M phosphate buffer (pH 7.4) for at least 2 h or overnight. Cells were pellets and kept in 8% (0.2 M) sucrose in 0.1 M phosphate buffer overnight at 4 °C (samples can be kept in sucrose for a long period of time). Cells were post-fixed with 1% OsO_4_ in 0.1 M phosphate buffer for 1 h and rinsed with 0.1 M phosphate buffer. The samples were dehydrated in increasing concentrations of ethanol and then infiltrated and embedded in LX-112 medium. The samples were then polymerized in a 60 °C oven for approximately three days. All reagents for fixation and processing were purchased from Sigma Chemical Co. (St. Louis, MO, USA). Ultrathin sections (0.5–1 µm) were cut using a Leica Ultracut microtome (Leica, Deerfield, IL, USA) and then stained with uranyl acetate and lead citrate. The stained samples were examined in a JEM 1011 transmission electron microscope (JEOL USA, Inc., Peabody, MA, USA). Digital images were obtained using an AMT imaging system (Advanced Microscopy Techniques Corp., Danvers, MA, USA).

### 4.5. Real-Time Polymerase Chain Reaction (qPCR)

We used qPCR to measure mRNA expression of metabolism-associated genes. The detailed protocol was published previously [37]. In brief, MCF-12A cells were cultured in media with 94 µg/mL azithromycin at 37 °C and 5% CO_2_ for 7 days. Cells cultured in the antibiotic-free media were used as controls. Cells were removed from plates by trypsin-EDTA digestion. Total RNA was isolated by PurLink RNA Kit (Invitrogen, Carlsbad, CA, USA). The cDNA was synthesized by the High Capacity RNA-to-cDNA kit (Applied Biosystems, Grand Island, NY, USA). Total RNA of 2 µg was mixed with 10 µL of 2× RT buffer and 1 µL of 20× Enzyme Mix and water for a total of 20 µL of reaction volume. The reaction mixture was incubated for 60 min at 37 °C and then 5 min at 95 °C to stop the reaction. The cDNA was ready for use in real-time PCR application or long-term storage in a freezer. We examined hypoxia-inducible factor alpha (*HIF1a*), three glycolytic enzymes (i.e., hexokinase (*HK2*), phosphofructokinase-1 (*PFKM*) and pyruvate kinase (*PKM2*)), and glucose transporter 1 (*SLC2A1*) and 3 (*SLC2A3*), and lactate dehydrogenase A (*LDHA*) which catalyzes the reduction of pyruvate by NADH to form lactate. The expression of mitochondrial biogenesis and antioxidant genes also was measured. Mitochondrial biogenesis genes included peroxisome proliferator-activated receptor-gamma coactivator 1 alpha (*PGC-1a*), nuclear respiratory factor 1 and 2 (*NRF1* and -*2*), and mitochondrial transcription factor A (*TFAM*). Antioxidant genes included superoxide dismutase (*SOD1, SOD2, SOD3*), catalase (*Cat*, glutathione peroxidase (*GPX1*), and thioredoxin-dependent peroxide reductase (*PRDX3*). All gene expression quantification was performed with the TaqMan Gene Expression Assay, a proven 5′ nuclease-based real-time PCR chemistry method. Primers and probes (PrimeTime Mini qPCR assay) were synthesized by Integrated DNA Technologies (IDT, Coralville, Iowa) (Table 4). β-actin (ACTB) was used as an endogenous gene control to normalize PCRs for the amount of RNA added to the reverse transcription reactions. Probes contained at the 5′ end the FAM (6-carboxy fluorescein) as a fluorescent reporter dye, and internal and at 3′ end the ZEN™/Iowa Black FQ as fluorescent double quenchers. Forty microliters of qPCR reaction mixture contained 20 µL of TaqMan universal PCR master mix (Applied Biosystems, Grand Island, NY), 4 µL of 10× PrimeTime Mini qPCR assay (IDT, Coralville, Iowa), and 16 µL of cDNA (100 ng). The qPCR reaction was aliquoted in triple to wells of a 384 well PCR plate. The plate was sealed, briefly centrifuged, and performed in reaction with a 7900HT real time PCR system (Applied Biosystems, Grand Island, NY). The standard mode ran for 2 min at 50 °C and 10 min at 95 °C, and 40 cycles (15 s at 95 °C and 1 min at 60 °C). Target gene expression was determined by relative quantification (RQ) which related the signal of the target transcript in a treated group to that of an untreated control (medium only). We analyzed relative quantification with the RQ Manager 1.2 software (Applied Biosystems, Grand Island, NY, USA). Gene expression was calculated as the ratio of mRNA of the cells cultured in 0.05 mg/mL gentamicin media to that of the cells in gentamicin free media. 

### 4.6. Measurement of Lactate 

Lactate concentration in culture media was measured with the Glycolysis Cell-Based Assay Kit (Cayman Chemical Company, Ann Arbor, MI, USA). The detailed method is found in the product protocol. Ten thousand MCF-12A cells or fibroblasts in 100 µL were sub-cultured in triple on a 96 well cell culture plate and cultured overnight at 37 °C and 5% CO_2_. The media were aspirated and replaced with 200 µL of 188 µg/mL of azithromycin in media. Control wells were filled with the media without any antibiotics. The plate was cultured at 37 °C for 24 h. Then, the plate was centrifuged at 1000 rpm for 5 min. Ten microliters of the supernatant from each well was transferred to corresponding wells on a new 96 well plate for lactate measurement. One hundred microliters of the Reaction Solution was added to each well, including the standard wells. The plate was incubated with gentle shaking on an orbital shaker for 30 min at room temperature. The absorbance was read at 492 nm with a plate reader. A standard curve was plotted using the absorbance of a series of standard. The lactate concentration of samples was calculated against the standard curve.

### 4.7. Mitochondrial Superoxide Detection

Mitochondrial superoxide was detected using the MitoSox Red mitochondrial superoxide indicator (Invitrogen, Carlsbad, CA, USA). The MitoSox Red reagent is live cell permeant and is rapidly and selectively targeted to the mitochondria. Once in the mitochondria, MitoSox Red reagent is oxidized by superoxide and exhibits red fluorescence. Ten thousand cells were cultured in 35 mm 1.5 glass bottom dishes containing gentamicin free media overnight. The media were aspirated on the second day. The dishes were filled with media containing azithromycin. The control cells were cultured in media without any antibiotics. All cells were culture for 24 h and stained with 5 µM MitoSox Red reagent working solution for 10 min at 37 °C. The cells were washed 3 times with Hank’s balanced salt solution and obtained fluorescence with a Olympus IX83 fluorescent microscope. Three pictures were taken in different areas of each dish. All pictures were taken with 2 s exposure. Fluorescent intensity (FI) was measured with the software Cellsens 1.16. Mean and standard deviation of the intensity was calculated.

### 4.8. DNA Oxidative Damage Examination

Ten thousand MCF-12A cells or fibroblasts in 100 µL were sub-cultured in triple on a 96 well cell culture plate and cultured for overnight at 37 °C and 5% CO_2_. The media were aspirated and replaced with 200 µL media containing azithromycin. Control wells were filled with the media without any antibiotics. The plate was then cultured at 37 °C for 24 h. The DNA oxidative damage in the culture supernatants was examined by DNA Damage Competitive ELISA Kit (Invitrogen, Carlsbad, CA, USA). Among the numerous types of oxidative DNA damage, the formation of 8-OHdG is a ubiquitous marker of oxidative stress. The 8-OHdG was quantitated by the DNA Damage Competitive ELISA Kit. In brief, all components were allowed to reach room temperature before use. Then, 50 µL of standard samples or cell culture media were added to the appropriate wells of the 96 well plate pre-coated with goat anti-rabbit IgG, as well as 25 µL of 8-OHdG and 25 µL of 8-OHdG antibody. The plate was covered with a plate sealer and incubated for 2 h at room temperature with shaking. The solution was aspirated and the wells washed 4 times with 300 µL of 1× washing buffer. The aspirate was thoroughly washed with washing buffer and 100 µL TMB substrate was added to each well. The plate was incubated for 30 min at room temperature without shaking. Then, 50 µL of Stop Solution was added to each well and the absorbance at 450 nm within 10 min was read. The concentration of 8-OHdG for each sample was calculated with the software Four Parameter Logistic Curve. 

### 4.9. Statistical Analysis

Data were analyzed by *t*-test and presented as mean ± standard deviation (SD). Findings were considered significant at *p* < 0.05.

## 5. Conclusions

Azithromycin induced mammalian mitochondrial toxicity, ROS production, DNA damage, aerobic glycolysis, and upregulation of the *HIF1a* gene which may contribute to tumorigenesis and neurodegeneration.

## Figures and Tables

**Table 1:** Lactate production of cell lines cultured in the media without or with azithromycin addition. L-lactate concentration (µM) was measured by the Cayman glycolysis cell-based assay kit.

	MCF-12A	Fibroblast
Cell number	10,000	10,000
Control (media without any antibiotic)	2.29 ± 0.41 (3) ^1^	3.01 ± 0.65 (3)
47 µg/mL azithromycin	3.03 ± 0.64 (3) NS ^2^	3.51 ± 0.59 (3) NS
94 µg/mL azithromycin	3.45 ± 0.56 (3) *p* < 0.05	4.38 ± 0.54 (3) *p* < 0.05
188 µg/mL azithromycin	3.91 ± 0.49 (3) *p* < 0.05	4.68 ± 0.75 (3) *p* < 0.05

^1^ Mean ± SD (*N*). ^2^ Unpaired *t*-test, less than 0.05 as significant. NS: not significant.

**Table 2:** Fluorescent intensity of mitochondrial superoxide in MCF-12A cells and fibroblasts (3 h of incubation). Mitochondrial superoxide was detected by MitoSOX red mitochondrial superoxide indicator.

	MCF-12A	Fibroblast
Control	25.5 ± 4.8 (3) ^1^	19.6 ± 5.6 (3)
Azithromycin 188 µg/mL	57.4 ± 13.6 *p* < 0.05 ^2^	45.3 ± 10.5 (3) *p* < 0.05
Azithromycin 188 µg/mL +SOD 100 u/mL	35.3 ± 5.4 NS	27.8 ± 8.9 (3) NS

^1^ Mean ± SD (*N*). ^2^ Unpaired *t*-test, compared to the control, less than 0.05 as significant. NS: not significant.

**Table 3:** Azithromycin caused DNA oxidative damage of MCF-12A cells and fibroblasts. The concentration of 8-HOdG (pg/mL) in the media was measured by the DNA Damage Competitive assay kit.

	MCF-12A	Fibroblast
Cell amount	10,000	10,000
Control (media without any antibiotic)	248.2 ± 65.6 (6) ^1^	388.0 ± 33.2 (3)
23.5 µg/mL azithromycin	295.7 ± 60.9 (3) NS ^2^	330.3 ± 117.6 (3) NS
94 µg/mL azithromycin	355.0 ± 29.6 (3) *p* < 0.05	331.3 ± 32.0 (3) NS
376 µg/mL azithromycin	889.0 ± 61.5 (3) *p* < 0.01	497.7 ± 48.5 (3) *p* < 0.05

^1^ Mean ± SD (*N*). ^2^ Unpaired *t*-test, compared to the control, less than 0.05 as significant. NS: not significant.

**Table 4:** List of primer and probe sequences for RT-qPCR.

Gene	Pair of Primers (FWD and REV)	Probe
*ACTB*	GGATCAGCAAGCAGGAGTATG;	AGAAAGGGTGTAACGCAACTAA
TCGTCCACCGCAAATGCTTCTAGG		
*HIF1a*	GTCTGCAACATGGAAGGTATTG;	GCAGGTCATAGGTGGTTTCT
ACTGCACAGGCCACATTCACGTAT		
*HK2*	GCAGAAGGTTGACCAGTATCTC;	CCAAGCCCTTTCTCCATCTC
CACATGCGCCTCTCTGATGAGACC		
*PFKM*	GCATCCCATTTGTGGTCATTC;	GTCACAGGTTGTGCAGATAGT
AATGTCCCTGGCTCAGACTTCAGC		
*PKM2*	CTGTGGCTGGACTACAAGAA;	CTGCTTCACCTGGAGAGAAATA
AAGTGGGCAGCAAGATCTACGTGG		
*SLC2A1*	CTGGGCAAGTCCTTTGAGAT;	GTGACACTTCACCCACATACA
AGTACACACCGATGATGAAGCGGC		
*SLC2A3*	AGGATGCAGGTGTTCAAGAG;	GCCCTTTCCACCAGAAATAGA
CGGCGCGGGTGTGGTTAATACTAT		
*LDHA*	AGATTCCAGTGTGCCTGTATG;	ACCTCTTTCCACTGTTCCTTATC
AGTGGAATGAATGTTGCTGGTGTCTCT		
*PGC-1a*	AGAGCGCCGTGTGATTTAT;	CTCCATCATCCCGCAGATTTA
ACCTGACACAACACGGACAGAACT		
*NRF1*	GTATCTCACCCTCCAAACCTAAC;	CCAGGATCATGCTCTTGTACTT
TGCAGCACCTTTGGAGAATGTGGT		
*NRF2*	GCAATCTGCTACACCTACTACC;	TCCCAGGTGAGCTTCTATCT
AAGCAGCCAAAGTACAAAGAGCGC		
*TFAM*	GTTGGAGGGAACTTCCTGATT;	CTGACTTGGAGTTAGCTGTTCT
AAGATGCTTATAGGGCGGAGTGGC		
*SOD1*	GTGCAGGGCATCATCAATTTC;	GGCCTTCAGTCAGTCCTTTAAT
AAGTAATGGACCAGTGAAGGTGTGGG		
*SOD2*	GCCTACGTGAACAACCTGAA;	GAAGAGCTATCTGGGCTGTAAC
TCACCGAGGAGAAGTACCAGGAGG		
*SOD3*	GCTGGCGCTACTGTGTT;	CGTGACCTTGGCGTACAT
AACTCTGACTCGGCGGAGTGGATC		
*CAT*	CTGGAGCACAGCATCCAATA;	TCATTCAGCACGTTCACATAGA
ATTGGCAGTGTTGAATCTCCGCAC		
*GPX1*	CAGTCGGTGTATGCCTTCTC;	GCTCGTTCATCTGGGTGTAG
TATCGAGAATGTGGCGTCCCTCTGA		
*PRDX3*	CCTTTGTGTGTCCTACAGAAATTG;	TCCAGGCAAGATGGCTAAAG
TGTGAAGTTGTCGCAGTCTCAGTGG		

## Supplementary Materials

The following are available online at https://www.mdpi.com/2079-6382/8/3/110/s1. Figure S1: Azithromycin upregulated gene expression of mitochondrial OXPHOS enzymes, mitochondrial biogenesis factors, antioxidants, *HIF1a,* and glycolytic enzymes in human fibroblasts; Figure S2: Azithromycin suppressed mitochondrial membrane potential and increased mitochondrial ROS of the mouse motor neuron cell line NSC34.
