# Supplementary material for: Mitochondrial Toxicity of Azithromycin Results in Aerobic Glycolysis and DNA Damage of Human Mammary Epithelia and Fibroblasts
Source: Antibiotics (Basel). 2019 Aug 3;8(3):110. doi: 10.3390/antibiotics8030110 (PMC6784251; doi:10.3390/antibiotics8030110)

A. Azithromycin upregulated gene expression of mitochondrial OXPHOS enzymes in fibroblasts. mRNA was measured by qPCR (The details in the Materials and Methods).

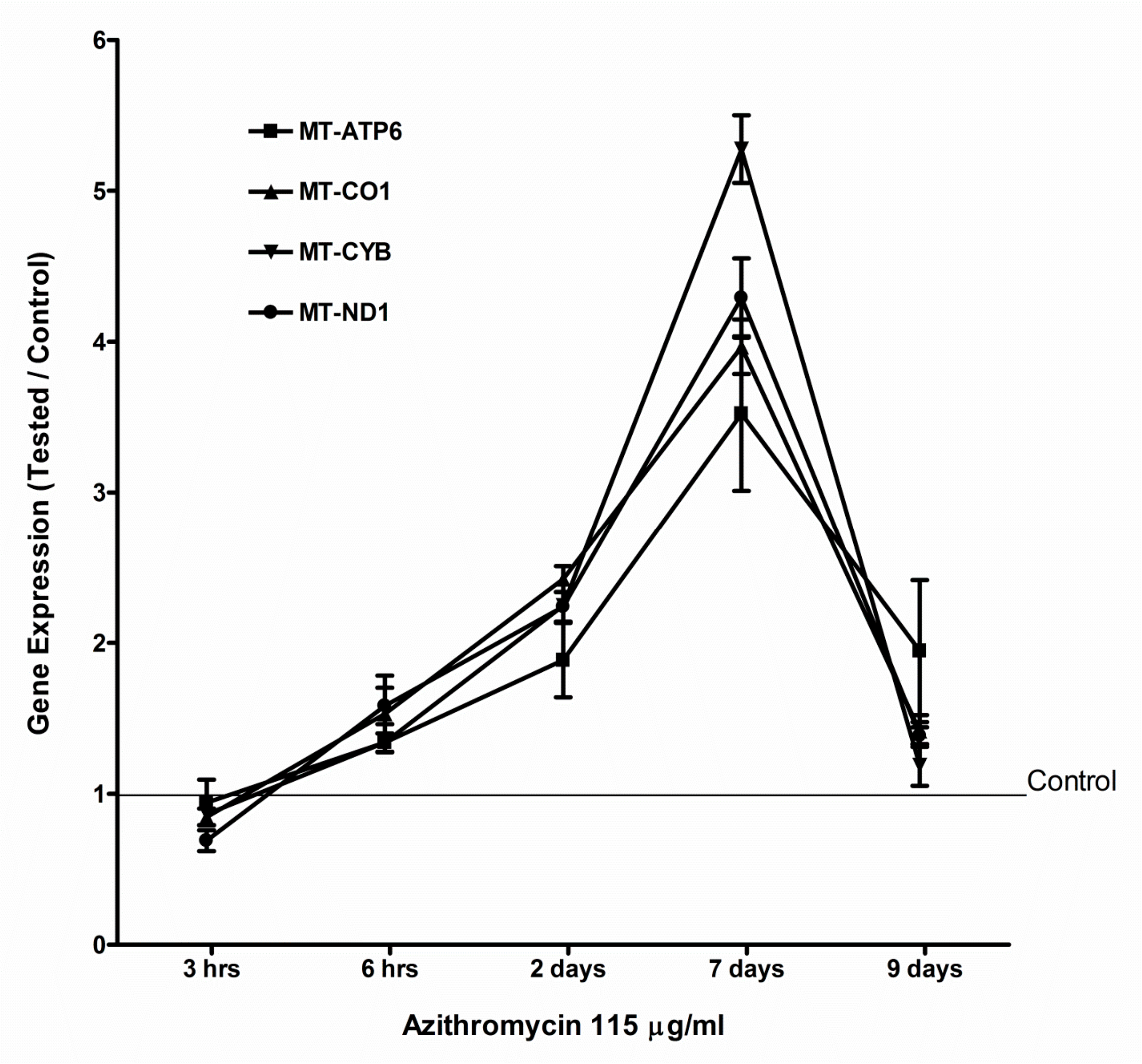

B. Azithromycin increased gene expression of mitochondrial biogenesis factors in fibroblasts. mRNA was measured by qPCR (The details in the Materials and Methods).

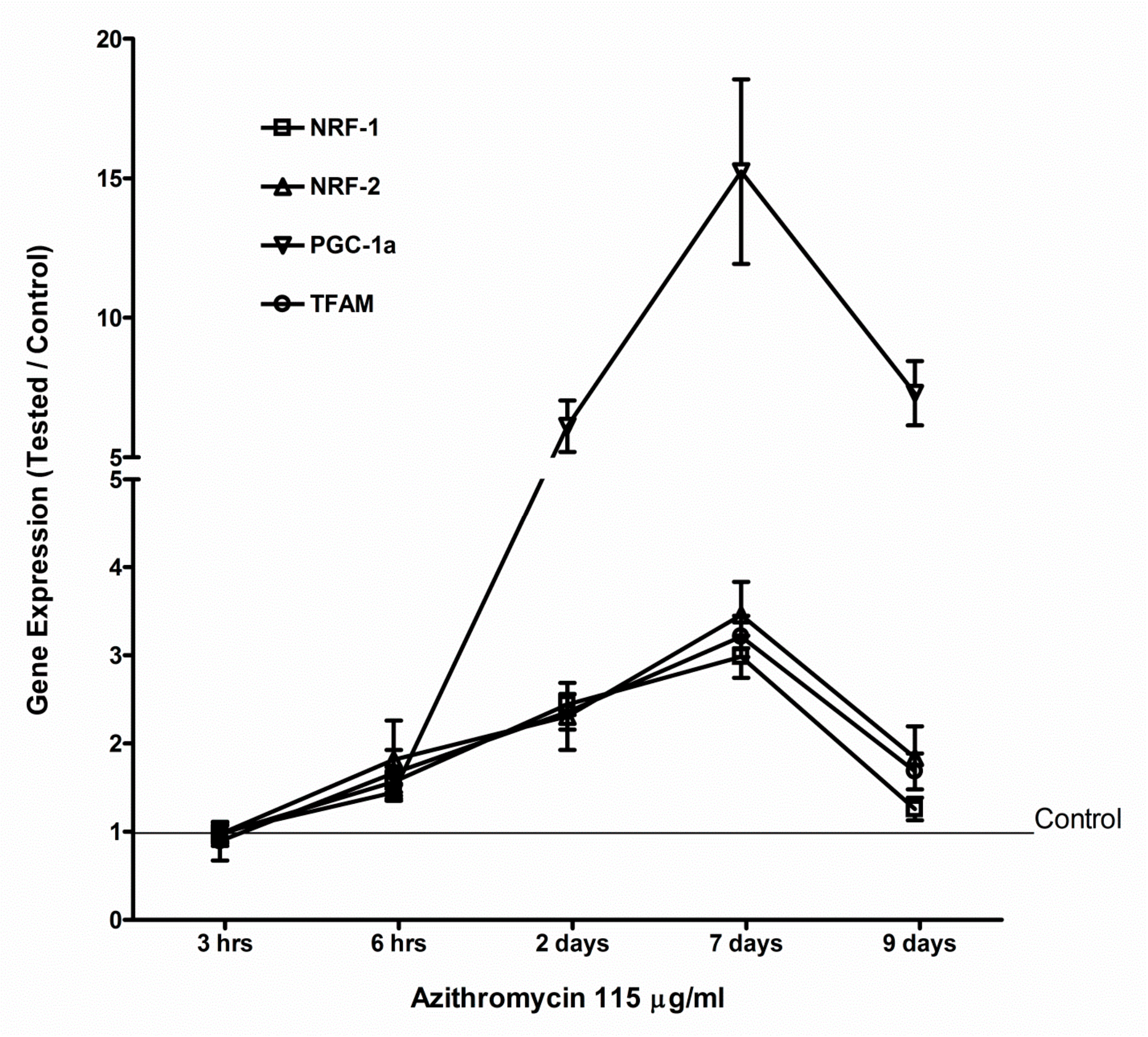

C. Azithromycin upregulated the expression of antioxidant genes in fibroblasts. mRNA was measured by qPCR (The details in the Materials and Methods).

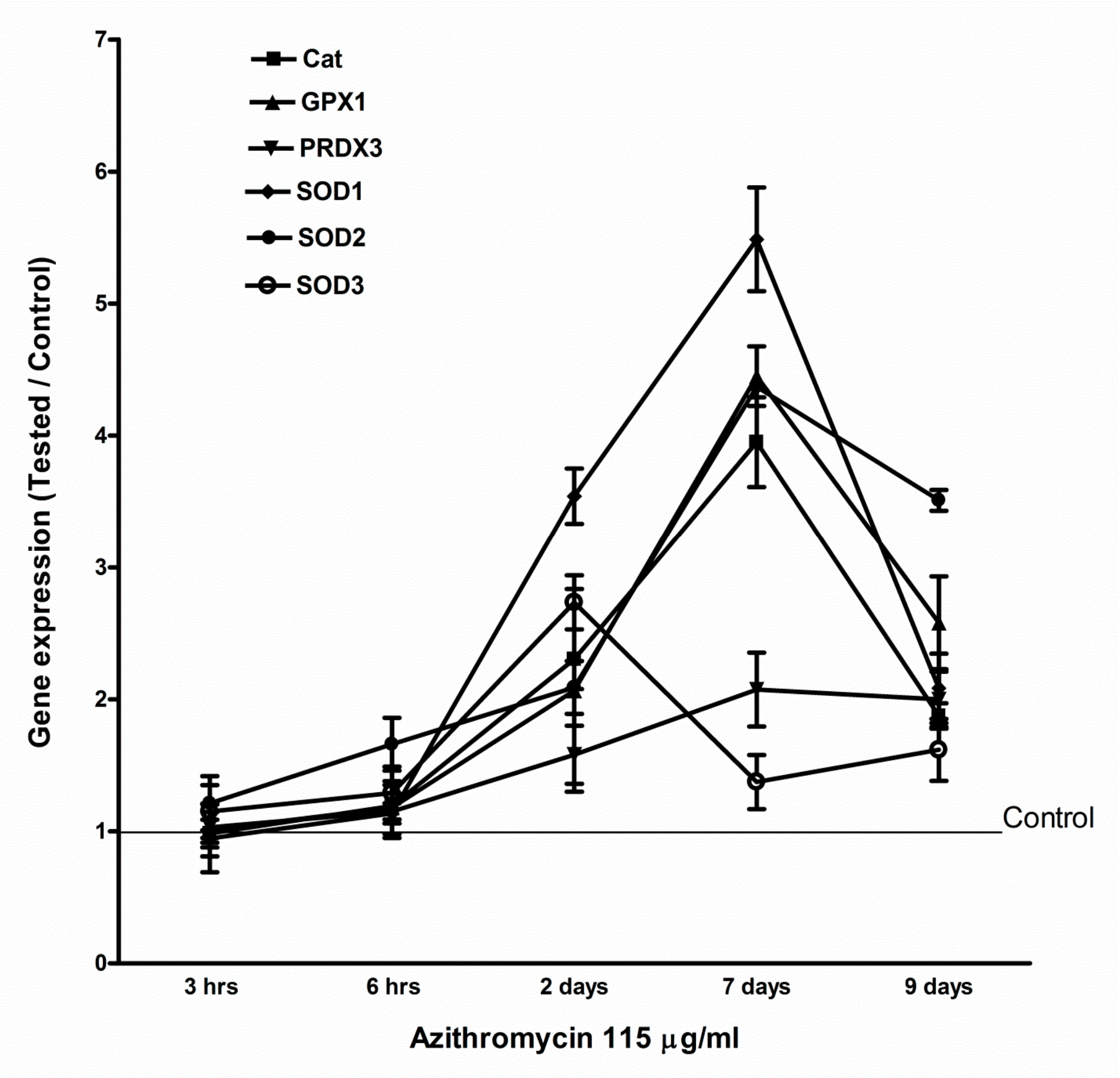

D. Azithromycin upregulated gene expression of HIF1a and glycolysis associated enzymes in fibroblasts. mRNA was measured by qPCR (The details in the Materials and Methods).

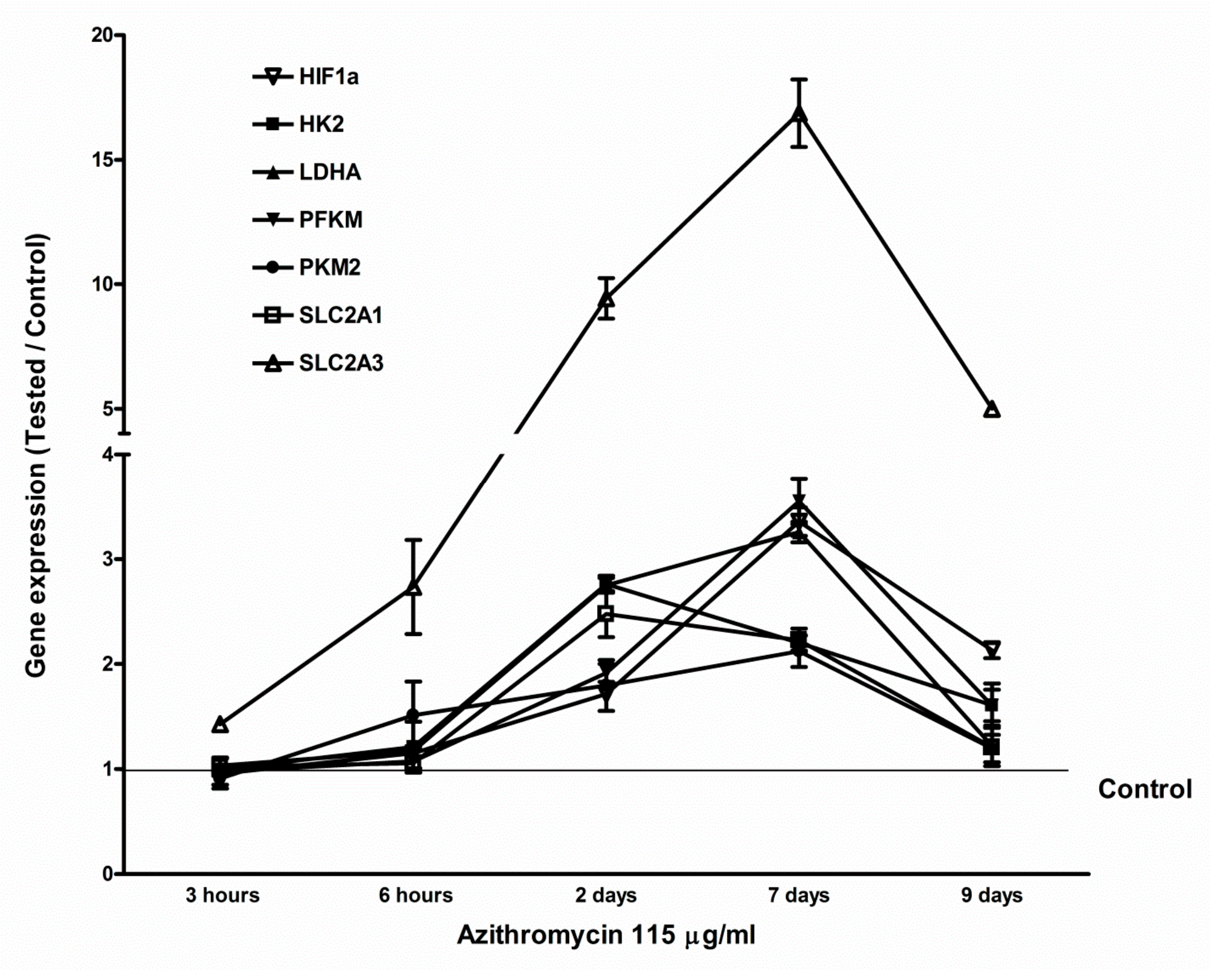

Figure S2--NSC34

A. Azithromycin suppressed mitochondrial membrane potential of mouse motor neuron cell line NSC34. a,c: phase contrast; b,d: fluorescence. a,b: control; c,d: incubated in the medium with 94μg/ml azithromycin for 3 hours.

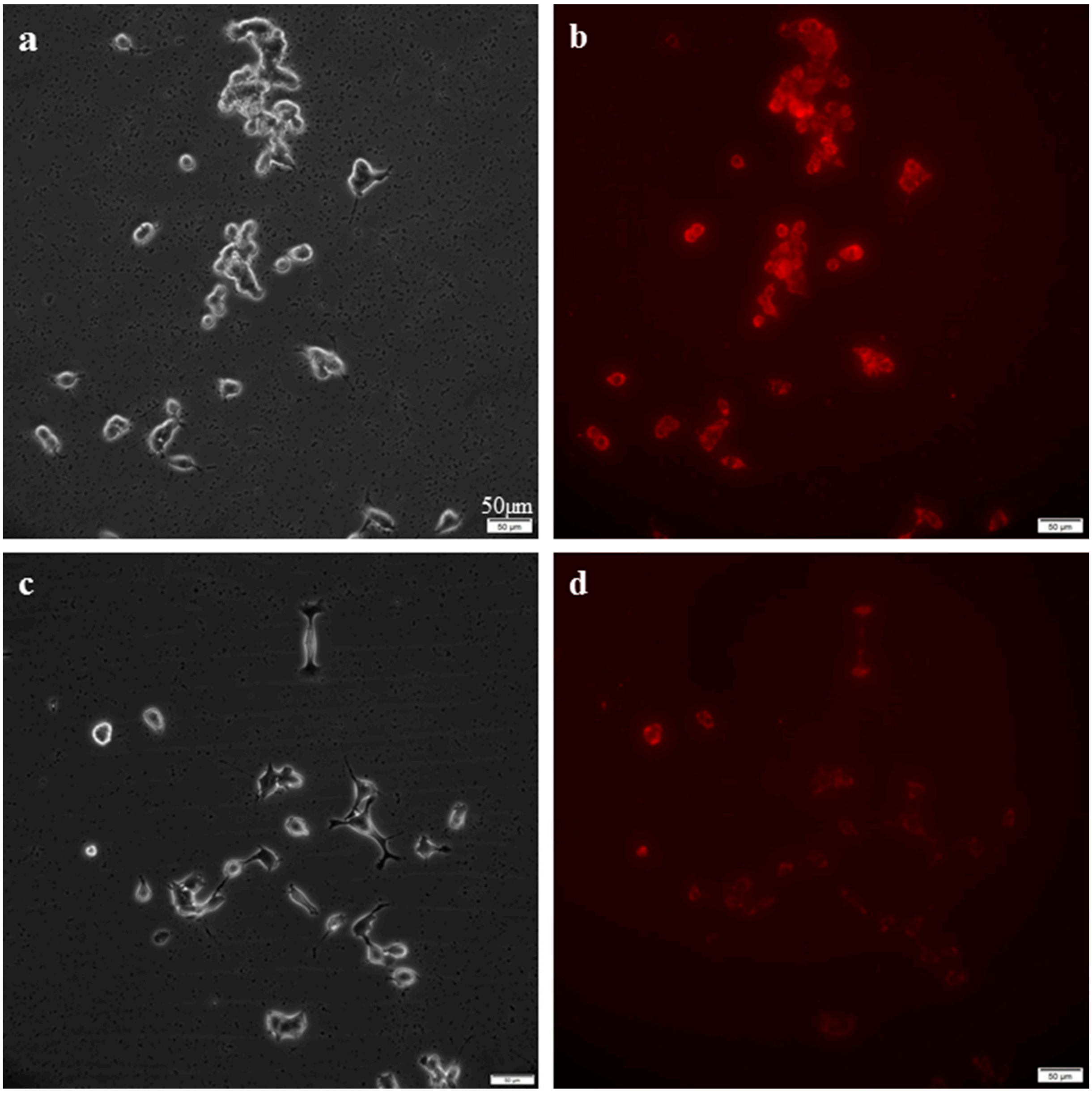
a
b
c
d
50μm
50 μm
50 μm
50 μm

B. Azithromycin increased mitochondrial ROS in NSC34 cells. a,c: phase contrast; b,d: fluorescence. a,b: control; c,d: incubated in the medium with 94μg/ml azithromycin for 3 hours.

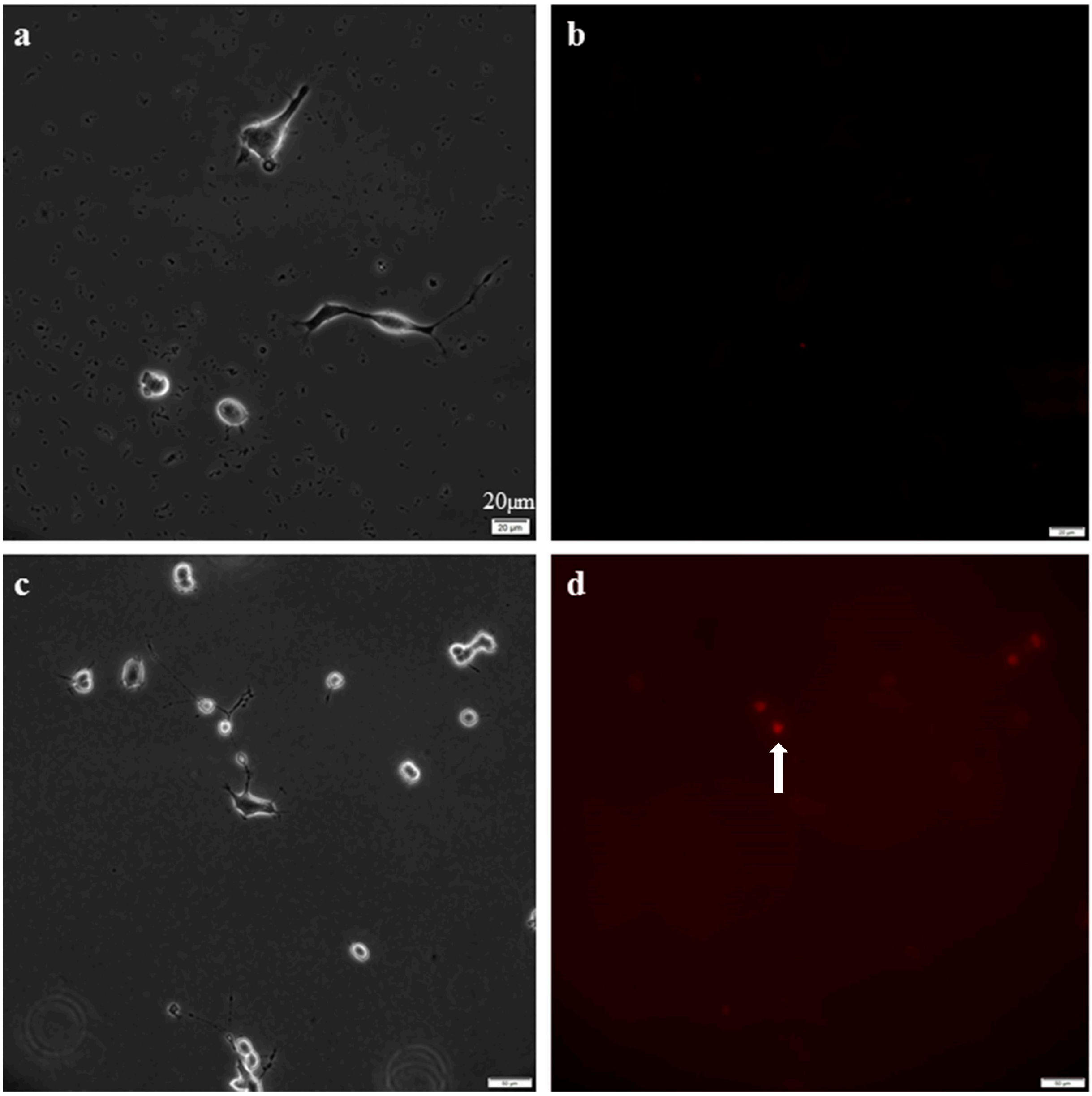

Supplement: Supplementary file 1 [file antibiotics-08-00110-s001.pdf]
